# Treatment of celiac artery rupture with a hybrid procedure involving aortic stent grafting and open surgery in a patient with neurofibromatosis type 1

**DOI:** 10.1016/j.jvscit.2022.07.020

**Published:** 2022-08-12

**Authors:** Yoshiaki Takata, Keijiro Katayama, Haruna Shimizu, Risa Inoue, Taiichi Takasaki, Shinya Takahashi

**Affiliations:** Department of Cardiovascular Surgery, Hiroshima University Hospital, Hiroshima, Japan

**Keywords:** Celiac artery rupture, Neurofibromatosis type 1, Hybrid treatment, EVAR treatment, Laparotomy

## Abstract

Neurofibromatosis type 1 is associated with vascular fragility, and vascular disease is the second leading cause of death in these patients. A 42-year-old woman with neurofibromatosis type 1 was transferred to our hospital owing to shock. A computed tomography scan revealed a ruptured celiac artery aneurysm, which had expanded from 14 to 26 mm in 1 day. The survival rate of patients with celiac artery rupture is extremely low, and there is no consensus on treatment. Here, we successfully performed a hybrid procedure with emergent implantation of aortic stent grafts for life-saving treatment and subsequent laparotomy for complete hemostasis.

Neurofibromatosis type 1 (NF1) is an autosomal-dominant genetic disorder and associated arterial rupture is rarely reported; however, systemic tissue and vascular fragility is observed in patients with NF1.^1^^,^^2^ Among visceral artery aneurysms (VAAs), rupture of celiac artery aneurysms (CAAs) is rare and few cases have been reported.^3^^,^^4^ There are reports of life-saving intervention for ruptured abdominal aneurysms, but no standard treatment methods have been established and the survival rate is extremely low. Here, we report a case of a ruptured celiac artery (CA) complicated by NF1 that was successfully treated using a hybrid procedure with emergent implantation of aortic stent grafts followed by open surgery. Patient consent was obtained for this case report.

## Case report

**Fig 1:**
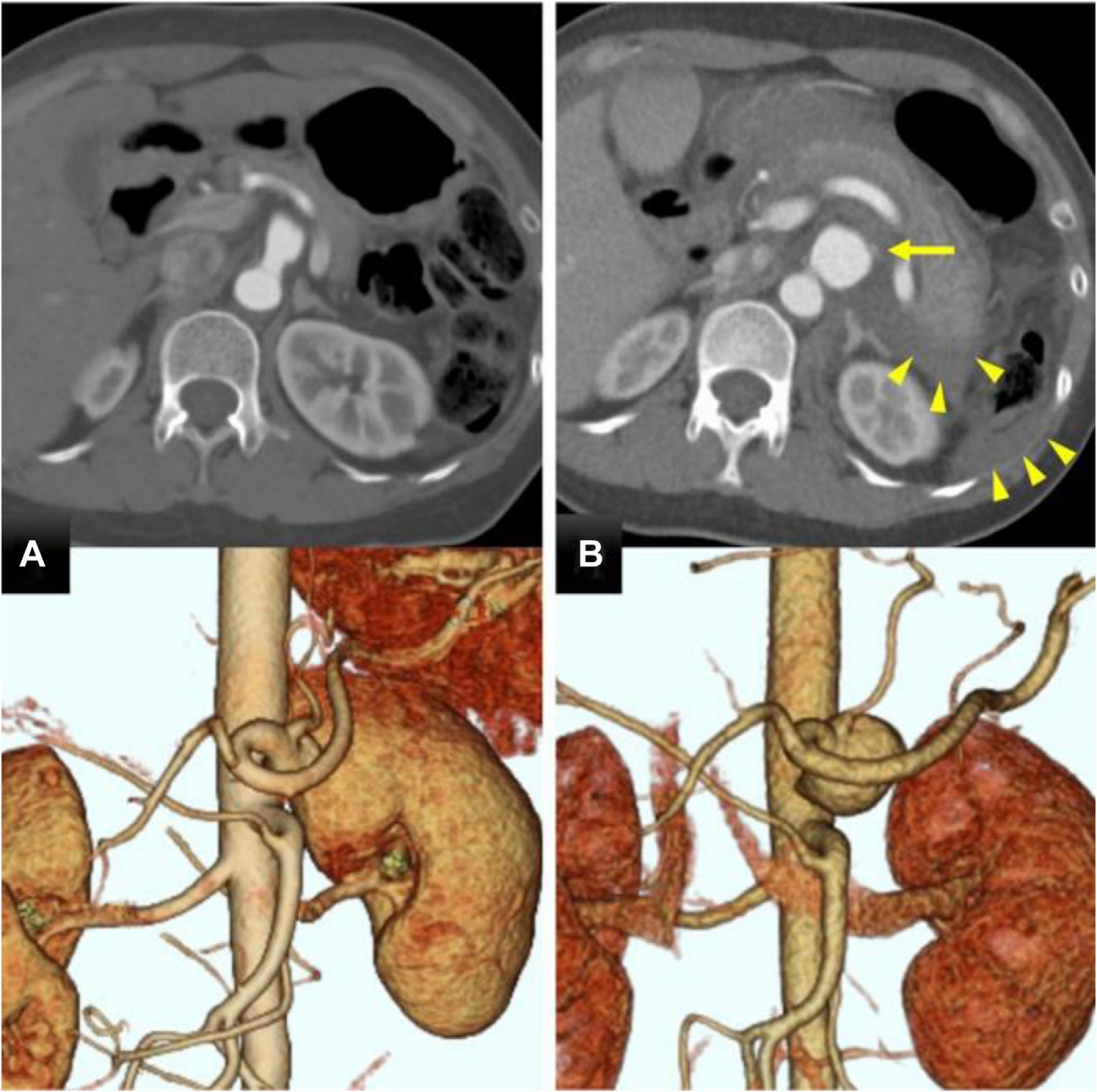
**A,** Computed tomography (CT) scan revealed a celiac artery aneurysm (CAA) with a diameter of 14 mm, immediately after the aortic bifurcation. **B,** The aneurysm expanded to 26 mm in just 1 day, and extravasation *(arrow)* and intra-abdominal fluid accumulation *(arrowheads)* were observed.

A 42-year-old woman with a history of NF1, antiphospholipid antibody syndrome, cerebral infarction, and deep vein thrombosis, who was on anticoagulation therapy, presented with back pain. A computed tomography (CT) scan revealed a CAA with a diameter of 14 mm (Fig 1, *A*), and she was admitted for conservative treatment. On the next day, her back pain worsened, and she exhibited tachycardia (heart rate, 116 bpm) and hypotension (blood pressure, 58/38 mm Hg). A complete blood count revealed a hemoglobin value of 5.6 g/dL, and a CT scan revealed an enlarged aneurysm with a diameter of 26 mm, active extravasation from the aneurysm, and intra-abdominal fluid accumulation (Fig 1, *B*). The rupture site was the CA, immediately after the origin of the abdominal aorta. She was subsequently transferred to our hospital.

**Fig 2:**
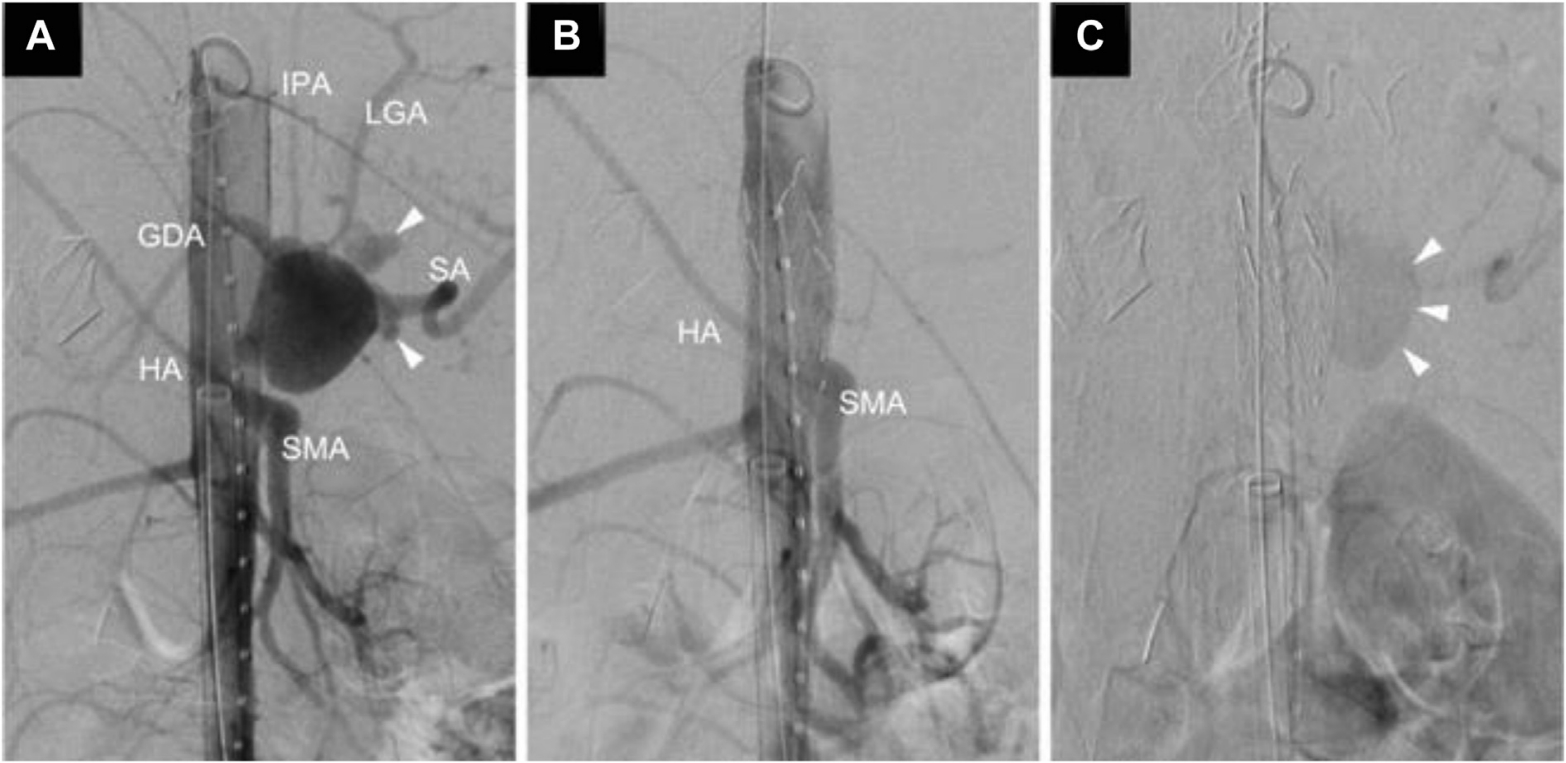
**A,** Aortography revealed a celiac artery aneurysm (CAA) with extravasation from its left side *(arrowheads)*. **B,** Aortic stent grafts were implanted immediately above the SMA. The hepatic artery originated from the SMA. **B** and **C,** Aortography revealed no evidence of contrast medium extravasation, but contrast from the visceral collateral circulation was still present *(arrowheads)*. *HA*, Hepatic artery; *GDA*, gastroduodenal artery; *IPA*, inferior phrenic artery; *LGA*, left gastric artery; *SA*, splenic artery; *SMA*, superior mesenteric artery.

**Fig 3:**
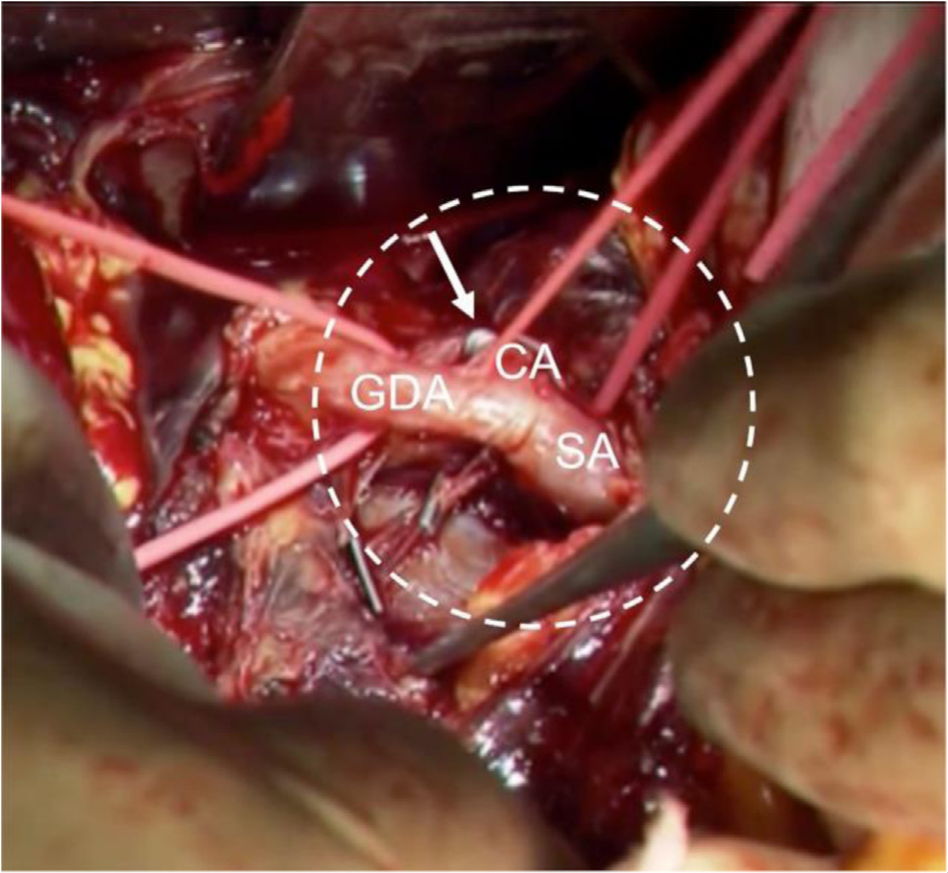
Operative findings of the aneurysm *(dotted circle)*. The outflows of the aneurysm were clipped *(arrow)* and the aneurysm was obliterated by oversewing with 5-0 polypropylene suture and felt strips at the root of the aneurysm. The GDA and SA arose aberrantly from the CA. *CA*, Celiac artery; *GDA*, gastroduodenal artery; *SA*, splenic artery.

She underwent an emergency hybrid treatment with abdominal aortic stent grafts (W. L. Gore & Associates, Flagstaff, AZ) and open surgery. Through the bilateral femoral arteries, an Aorta Extender 26 mm × 3.3 cm (Excluder PLA260300J, W. L. Gore & Associates) and 28.5 mm × 3.3 cm (Excluder PLA280300J, W. L. Gore & Associates) were implanted immediately above the superior mesenteric artery (SMA) to close the CA origin (Fig 2, *A*). Aortography revealed no evidence of contrast medium extravasation (Fig 2, *B*). She was stabilized hemodynamically; however, there was persistent back bleeding from the branches of the CA (Fig 2, *C*). A laparotomy was performed through a midline abdominal incision from the xiphoid process to the lower abdomen, and the distal CA, left gastric artery, and inferior diaphragmatic artery were clipped (Fig 3). Minor hemorrhage was observed deep within the aneurysm, which may have been due to endoleak from the stent grafts. The aneurysm was obliterated by oversewing with 5-0 polypropylene suture and felt strips to achieve hemostasis. Aortography demonstrated no further extravasation of blood from the aneurysm. The SMA and hepatic artery, which arose aberrantly from the SMA, were patent, and the gastroduodenal artery and splenic artery maintained blood flow from the pancreaticoduodenal arcade artery. Thus, we did not bypass the CA. The operative time was 218 minutes.

On postoperative day (POD) 1, a CT scan revealed extravasation from the jejunal branch of the SMA, which was not directly manipulated during the operation. It was embolized with 0.3 mL of *N*-butyl-2-cyanoacrylate (NBCA) mixed with ethiodized oil (Lipiodol) in a 1:3 ratio through an endovascular microcatheter. The procedure was completed without any apparent extravascular leakage. On POD 2, the patient developed anemia and abdominal distension. CT findings showed hemorrhage from a branch of the right hepatic artery that arose from the SMA and an intra-abdominal hematoma. We managed to embolize it with 0.2 mL of NBCA mixed with lipiodol in a 1:2 ratio and a piece of gelatin sponge (Serescue). No extravascular leakage was subsequently observed. The hemorrhages were presumed to have been iatrogenic and associated with vascular fragility owing to the NF1.

**Fig 4:**
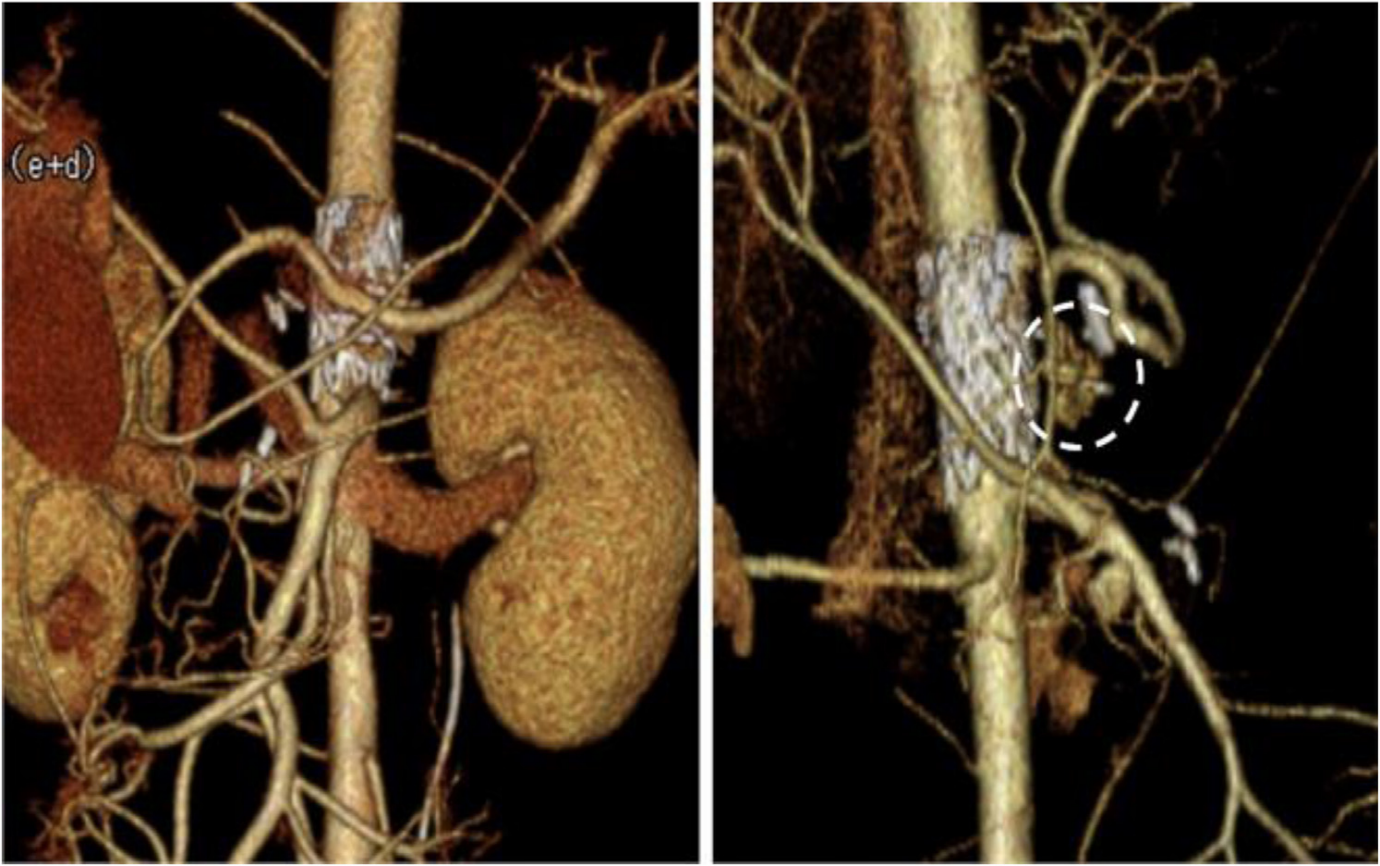
A postoperative computed tomography (CT) scan revealed that the celiac artery aneurysm (CAA) was treated at its origin and had disappeared *(dotted circle)*. Blood flow in the celiac artery branches was maintained by the pancreaticoduodenal artery arcade.

Postoperatively, the patient developed renal insufficiency and liver damage owing to abdominal compartment syndrome, which reached a pressure of 24 mm Hg and required abdominal catheter drainage and dialysis. On POD 20, the patient was transferred to a referral hospital for further treatment and rehabilitation. Dialysis was discontinued and the tracheostomy was closed on POD 91. A postoperative CT scan revealed that the CAA was treated at its origin and that blood flow through the gastroduodenal artery and splenic artery was maintained by the pancreaticoduodenal arcade artery (Fig 4). She was followed up for 1 year without any major problems.

## Discussion

NF1 causes pigmented skin lesions, multiple neurofibromas, and various lesions in the nerves, bones, eyes, and blood vessels.^5^ This patient was diagnosed based on the presence of café-au-lait spots, neurofibromas, and family history. NF1 may be associated with vascular lesions, such as arterial stenosis, aneurysms, spontaneous rupture of arteries, and arteriovenous malformations, in 0.4% to 6.4% of cases.^6^ Patients with NF1 have a median life expectancy that is 8 to 15 years shorter than the general population. Moreover, vascular disease is the second leading cause of death in these patients.^7^^,^^8^ Arterial rupture in a patient with NF1 can be critical given the underlying vascular fragility.

VAAs are rare, with CAAs accounting for 4% of 30% of cases.^9^^,^^10^ CAA rupture occurs in 10% to 20% of patients and is associated with a minimum mortality rate of 50%.^11^, ^12^, ^13^ CAAs in patients with NF1 would be more likely to rupture and have higher mortality rate because of the vascular fragility. Endovascular treatment (EVT) for VAAs has several advantages over surgical therapy^14^, ^15^, ^16^, ^17^; however, EVT of CAAs is challenging because of the remaining outflows and the risk of ischemia of the abdominal organs. Moreover, CA rupture can cause rapid shock and requires immediate diagnosis and treatment. We believe that hybrid surgery, in which EVT and laparotomy are performed simultaneously, could be a useful treatment option. The most effective method of saving the patient's life is to close the inflow to the ruptured aneurysm by promptly inserting an aortic stent graft and bringing the patient out of shock. This strategy allows time for a laparotomy to close the outflow and bypass the visceral artery if necessary. For patients in shock, such as in this case, a treatment strategy involving prompt, life-saving stent grafting, followed by laparotomy for complete hemostasis and revascularization, if abdominal organ ischemia occurs, can be considered.

Based on the imaging review, the patient was considered to have a rapidly expanding and ruptured aneurysm. The Society for Vascular Surgery clinical practice guidelines recommend that all ruptured CAAs (class 1A), all CA pseudoaneurysms (class 1B), and greater than 2 cm CAAs (class 1C) be treated.^18^ CA rupture has been reported to occur in approximately 5% of CAAs ranging from 15 to 22 mm in diameter and in 50% to 70% of those greater than 32 mm.^19^^,^^20^ In this case, the diameter was only 14 mm at admission, suggesting that it was less likely to rupture and that treatment is not indicated. Conservative treatment may be considered in many cases, but in patients with fragile vessels, such as those with NF1, the risk of rupture may be high, and early intervention should be considered.

## Conclusions

We encountered a case of CA rupture associated with NF1, in which aortic stent grafting and surgical aneurysm closure were performed simultaneously with good results. Conservative management is the standard treatment for CAAs without significant enlargement; however, early intervention may be necessary for patients with vulnerable arteries, such as those with NF1. In time-sensitive scenarios, closure of the inflow could bring the patient out of shock and save their life, and our treatment strategy should be considered by physicians facing similar situations.
